# The cost of a meal: factors influencing prey profitability in Australian fur seals

**DOI:** 10.7717/peerj.12608

**Published:** 2021-12-08

**Authors:** Nelle Meyers, Cassie N. Speakman, Nicole A.S.-Y. Dorville, Mark A. Hindell, Jayson M. Semmens, Jacquomo Monk, Alistair M.M. Baylis, Daniel Ierodiaconou, Andrew J. Hoskins, Greg J. Marshall, Kyler Abernathy, John P.Y. Arnould

**Affiliations:** 1 School of Life and Environmental Sciences, Deakin University, Burwood, Victoria, Australia; 2 Flanders Marine Institute (VLIZ), Ostend, Belgium; 3 Institute for Agricultural and Fisheries Research (ILVO), Ostend, Belgium; 4 Department of Biology, Centre for Forest Interdisciplinary Research, University of Winnipeg, Winnipeg, Manitoba, Canada; 5 Institute for Marine and Antarctic Studies, University of Tasmania, Hobart, Tasmania, Australia; 6 South Atlantic Environmental Research Institute, Stanley, Falkland Islands; 7 CSIRO Health and Biosecurity, Townsville, Queensland, Australia; 8 Exploration Technology Lab, National Geographic Society, Washington D.C., United States of America

**Keywords:** Animal-borne video, Foraging efficiency, Marine predator, Optimal foraging, Prey energetics, Profitability, Crittercam, Benthic foraging

## Abstract

Knowledge of the factors shaping the foraging behaviour of species is central to understanding their ecosystem role and predicting their response to environmental variability. To maximise survival and reproduction, foraging strategies must balance the costs and benefits related to energy needed to pursue, manipulate, and consume prey with the nutritional reward obtained. While such information is vital for understanding how changes in prey assemblages may affect predators, determining these components is inherently difficult in cryptic predators. The present study used animal-borne video data loggers to investigate the costs and benefits related to different prey types for female Australian fur seals (*Arctocephalus pusillus doriferus*), a primarily benthic foraging species in the low productivity Bass Strait, south-eastern Australia. A total of 1,263 prey captures, resulting from 2,027 prey detections, were observed in 84.5 h of video recordings from 23 individuals. Substantial differences in prey pursuit and handling times, gross energy gain and total energy expenditure were observed between prey types. Importantly, the profitability of prey was not significantly different between prey types, with the exception of elasmobranchs. This study highlights the benefit of animal-borne video data loggers for understanding the factors that influence foraging decisions in predators. Further studies incorporating search times for different prey types would further elucidate how profitability differs with prey type.

## Introduction

Foraging success is one of the basic components of individual fitness, with direct impacts on individual reproductive success, population growth, and, ultimately, survival of a species (Schoener, 1971). Determining the factors shaping foraging behaviour of animals has long been an important aim in behavioural studies (Pyke, Pulliam & Charnov, 1977). Such information is central to understanding an animal’s role within the ecosystem and to predicting its responses to environmental variability. For animals to be optimal in their foraging choices, they should select prey which have the highest return for the least cost (Krebs & Davies, 2009; Pyke, Pulliam & Charnov, 1977; Schoener, 1971). Further, animals should prefer prey items that have the greatest profitability and ignore prey types of lower profitability unless there is limited availability of the preferred prey types (Goss-Custard, 1977).

An important factor influencing optimal diet choice is the time needed to process food items (*i.e.*, handling time). Handling time is the time needed to manipulate and consume a prey item, which influences the time remaining within a foraging trip to search for additional prey (Wolf, Hainsworth & Gill, 1975). Optimal diet choice also accounts for energetic constraints, such as the metabolic cost of the different foraging activities (Krebs & Davies, 2009). Ultimately, the nutritional reward an animal receives per unit time will influence its optimal diet choice. Prey profitability can be estimated by quantifying the energy gain from prey (influenced by size, morphology, and nutritional content) and energy expenditure for pursuit and handling durations (Stephens et al., 1986).

Most marine mammals forage at considerable depths, and often in remote locations (Bowen et al., 2002), making observations of the fine-scale aspects of their foraging behaviour logistically difficult. Consequently, while numerous studies have investigated the at-sea movements of marine mammals searching for prey (*e.g.*, Austin et al., 2006; Sims et al., 2008), little is known of their prey choices and foraging efficiency due to the difficulty of simultaneously measuring the prey field, energy gain and expenditure. Recent advances in animal-borne video data logger technology have enabled the study of fine-scale foraging behaviour in marine mammals while simultaneously observing the prey field (Bowen et al., 2002; Kernaleguen et al., 2016; Wilson et al., 2017), providing new opportunities for assessing prey profitability and the factors influencing it.

The Australian fur seal (*Arctocephalus pusillus doriferus*; AUFS) is the largest of the fur seal species, with adult females and males weighing on average 76 kg and 279 kg, respectively (Shaughnessy & Warneke, 1987). The species has a breeding distribution restricted to south-eastern Australia and forages almost exclusively within the shallow (60–80 m) continental shelf waters of Bass Strait and its approaches (Arnould & Hindell, 2001; Kirkwood & Arnould, 2011). Bass Strait is considered to be an area of low marine primary productivity, but oceanographic features, such as the adjacent seasonally active Bonney Upwelling and the South Australia Current, feed it with secondary productivity (Gibbs, Tomczak & Longmore, 1986; Kämpf, 2015; Sandery & Kämpf, 2007). Driven largely by the intensification of the East Australian Current, the region is one of the fastest warming oceanic areas in the Southern Hemisphere (Hobday & Lough, 2011; Hobday & Pecl, 2014). The anticipated oceanographic changes are predicted to greatly alter the abundance, distribution, and diversity of prey species (Hobday & Lough, 2011). Indeed, the increased southwards incursions of warm, nutrient poor water has already led to changes in many marine ecosystem communities (Hobday et al., 2006; Last et al., 2011; McLeod et al., 2012).

The AUFS is a generalist predator that forages predominantly on and near the seafloor (Arnould & Hindell, 2001; Speakman et al., 2020) and consumes a wide range (>60 species) of cephalopods, elasmobranchs, and bony fish (Deagle, Kirkwood & Jarman, 2009; Hume et al., 2004). While numerous studies have investigated the foraging behaviour of AUFS and the intrinsic and extrinsic factors influencing it (Arnould & Hindell, 2001; Hoskins & Arnould, 2013; Hoskins et al., 2015; Kirkwood & Arnould, 2011; Knox, Baylis & Arnould, 2017; Speakman et al., 2020), little is known of the determinants of prey choice and foraging efficiency in the species. Such knowledge is necessary for predicting how the species may respond to changes in the diversity, distribution, and abundance of their prey. This is crucial for understanding the impact of potential future environmental changes on the species’ reproductive success and, ultimately, their population trajectory (Costa et al., 2010).

Therefore, the aims of the present study were to use animal-borne video cameras to determine in female AUFS: (1) the energetic costs related to prey pursuit and handling; (2) the rate of energy expenditure and intake associated with different prey types; and (3) the profitability of different prey types.

## Materials and Methods

### Ethics statement

All research procedures were approved by the Deakin University Animal Ethics Committee (A16-2008, A14-2011, B16-2014) and under a Department of Land, Water Environment and Planning (Victoria, Australia) Wildlife Research Permits (10005484, 10007153, 1000826).

### Animal handling and instrumentation

The study was conducted at Kanowna Island, central northern Bass Strait, south-eastern Australia (39°10′S, 146°18′E). The island hosts the third largest breeding colony of AUFS, with an annual pup production of *ca* 2,500 (McIntosh et al., 2018). Between May-August during 2008–2017, adult females observed provisioning pups in the hinterland of the colony were selected haphazardly, captured with a modified hoop net (Fuhrman Diversified, Seabrook, Texas, USA) and anaesthetised using isoflurane delivered via a portable gas anaesthetic machine (Stinger, Advanced Anaesthesia Specialists) (Gales & Mattlin, 1998) to facilitate handling during procedures. Once anaesthetised, individuals were weighed on a platform using a digital suspension scale (±0.5 kg) and morphometric measurements (standard length, flipper length, axillary girth) were obtained using a tape measure (±0.5 cm).

A digital video data logger was then attached to the dorsal fur between the scapula using quick-setting epoxy (Accumix 268; Huntsmen, Texas, USA). Three video data logger models were used during the study: Crittercam (Gen 5.7; National Geographic Society, Washington, D. C., USA; 23 cm in length, 5.7 cm in diameter, 800 g); CATS-DC (v6-5.7.0, Customized Animal Tracking Solutions, Moffat Beach, Australia, 8.0 cm × 11.0 cm × 4.5 cm, 180 g) and BBC custom-built cameras (v1; Mr ROV, Bristol, UK, 13.0 cm × 5.0 cm × 4.5 cm, 240 g). The cameras were positioned so that the seal’s head was visible in the field of view. The distance between the top of the seal’s head (between the pinnae), the head width at the pinnae, and snout to the lens of the video camera was determined using a tape measure (±0.5 cm) for prey size estimation.

A dive behaviour data logger (MK10, Wildlife Computers, Washington, D.C., USA, 7.4 cm × 5.7 cm × 3.6 cm, 120 g) and/or GPS data logger (F1G, Sirtrack, Havelock North, NZ, 10.0 cm × 2.8 cm × 5.2 cm, 117 g) and a VHF transmitter (Sirtrack, Havelock North, NZ), to facilitate relocation for recapture, was glued to the dorsal pelage in series with the video data logger to minimise additional drag. Individually numbered plastic tags were inserted into the trailing edge of the fore flippers before the animal was allowed to recover from anaesthesia and resume normal behaviours. Animals were recaptured after one or more foraging trips to sea upon which the data loggers were removed by cutting the fur beneath the devices, and the data were downloaded to a portable computer.

The Crittercam video data loggers were programmed to record video on a duty cycle of 1 h on:3 h off starting at 6 am (AEST). To conserve battery life and to maximise the number of recorded dives, the video data logger started recording only when seals were submerged below 40 m and stopped recording once the seal ascended again above 40 m. The 40 m cut-off was used as AUFS are predominantly benthic foragers, foraging on the sea floor (mean depth 60–80 m) of Bass Strait, thus maximising battery life while minimising missed foraging dives. Night recordings were possible through near-infra red LED beams that illuminated the field of view up to 5 m in front of the seal. The CATS-DC and BBC video data loggers were programmed on a continuous recording schedule of 1 h and 2 h duration, respectively, at 10:00 (AEST) and 14:00 (AEST) each day once the animal entered the water (at all depths) until the battery power expired.

### Data processing

**Figure 1:**
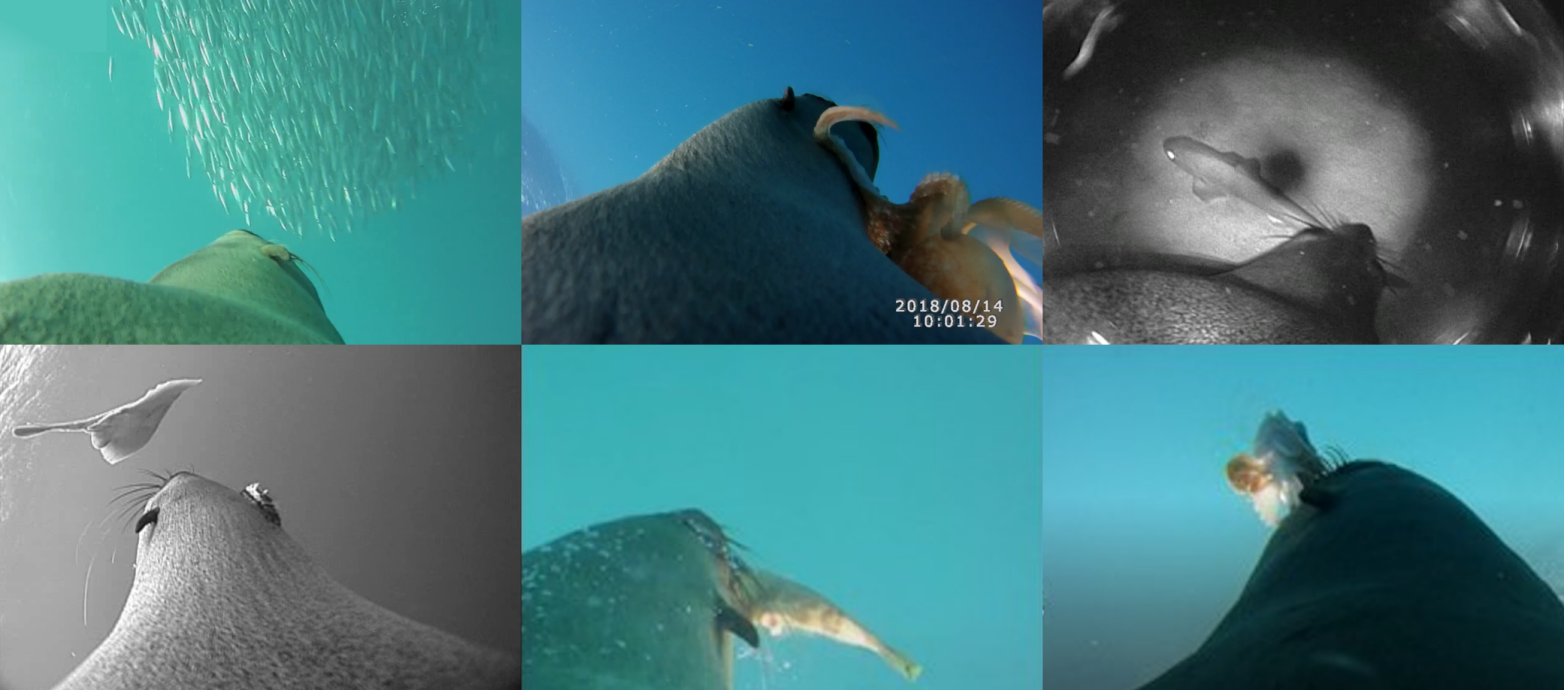
Representative images from animal-borne video data loggers showing captures of the various prey categories by female Australian fur seals. From left to right, upper to lower row: schooling jack mackerel (*Trachurus sp.*, Baitfish); an octopus (*Octopus sp*., Cephalopod); a dogfish (Squaliformes, Elasmobranch); a stingray (Myliobatiformes, Elasombranch); a leatherjacket (Tetraodontiformes, Solitary fish) and a gurnard (Scorpaeniformes, Solitary fish). Images are from animal-borne video cameras deployed by JPY Arnould.

Information from the video data loggers was processed frame-by-frame using the Solomon Coder Software version Beta 17.03.22 (Eötvös Loránd University, Budapest, Hungary). Information for each frame was categorized for the individual’s position in the water column (*i.e.*, surface, descending, transiting along the sea floor, and ascending), foraging behaviour (*i.e.*, pursuing prey, handling prey), and prey type and abundance (*i.e.*, solitary or schooling) present. Prey were identified to the lowest taxonomic level possible using a field guide (Kuiter, 1997) and identifications were confirmed by researchers experienced in video analyses of marine species (Fig. 1). With the exception of Scorpaeniformes, Tetraodontiformes, and Elasmoranchii, which could only be identified to the family level, all other prey types were identified to the genus or species level. Subsequently, prey items were categorized broadly into more general groups: ‘Benthic — Cephalopods’, ‘Benthic - Elasmobranchs’, ‘Benthic—Solitary fish’, ‘Demersal — Baitfish’ (schooling species usually associated with the water column but detected near the sea floor), ‘Pelagic — Baitfish’, and ‘Pelagic—Solitary fish’. Prey were classified as pelagic prey if they were detected in the midwater and benthic prey were classified as benthic prey when the detected prey item was visibly on or near the seafloor in the video footage (Fig. 1). When prey items could not be identified, which occurred only within the benthic zone, prey were characterised as ‘Benthic — Unknown’.

Prey detections refer to prey items observed in the video recordings with or without pursuit initiation (*i.e.*, the seal begins prey pursuit). A prey pursuit was defined as starting when the seal made intentioned movements (*i.e.*, lunged) towards a prey and ended once the prey was captured (if successful) or when the seal terminated the pursuit. The handling duration was defined as the time from when the seal captured the prey until the seal consumed the prey or discarded any remaining portion. The total time was defined as the pursuit time plus handling time. As the Crittercam data loggers recorded video only at depths below 40 m, the handling times could not be recorded for many larger prey that were not consumed on the sea floor.

Where possible, prey size was estimated from digital still images taken from the video data when the prey was at the tip of the seal’s snout or in its mouth using the equation:

(1) \begin{eqnarray*}By=[(Bx\div Bm)\cdot Am]\cdot (Bz\div Az)\end{eqnarray*}

where *B*_*y*_ is the estimated prey size (cm), *A*_*m*_ is the length of the prey (cm) on the image, *B*_*m*_ the width of the seal’s head (cm) on the image, *B*_*x*_ is the actual width of the seal’s head (cm), *A*_*z*_ is the distance (cm) between the camera lens and where the head width was measured, and *B*_*z*_ is the distance (cm) between where the head width was measured and the prey in the mouth (Carlbom & Paciorek, 1978). Both *A*_*z*_ and *B*_*z*_ allow for calculation of items at a distance from the camera lens by using reference items of known size in the video frame. Fishes and sharks were measured by their fork length (cm), stingrays by their total length (cm) and cephalopods by their mantle length (cm).

Estimated prey lengths were then converted to estimates of body mass (g) using published length-mass relationships for the observed, or a closely related, species (Table S1) (Alejo-Plata & Mendez, 2014; Lteif et al., 2016; Smallwood, Tate & Ryan, 2017; Vallisneri, Montanini & Stagioni, 2010). Estimates of the gross energy content of prey consumed were subsequently derived from published prey-specific energy densities (J g^−1^) (Pehrsson et al., 2015; Sidwell, 1981) and the estimated mass and the proportion of the prey consumed. The amount of energy expended by the seals during prey pursuit and handling durations was estimated, where possible, for each of the broad prey categories. The energy expended was estimated using the diving metabolic rate of captive Steller sea lions (*Eumetopias jubatus*) recorded by respirometry (47.6 mLO_2_ kg^−1^ min^−1^) (Volpov et al., 2015) combined with pursuit and handling durations of different prey types obtained in this study for the seal individuals.

The profitability (kJ s^−1^) of each prey type was determined by the foraging efficiency with which they were consumed using the equation (Stephens & Krebs, 1986):

(2) \begin{eqnarray*}\text{Prey profitability} = \frac{{\mathrm{GE}}_{\text{intake}}-{\mathrm{GE}}_{\text{expended}}}{\text{pursuit}+\text{handling time}} \end{eqnarray*}

where GE_intake_ represents the gross energy intake (kJ) and GE_expended_ represents the energy expended (kJ) during prey pursuit and handling (s). Prey profitability was calculated for each individual successful prey capture. Ordinarily search time is incorporated in calculations of prey profitability (Stephens et al., 1986). However, search time is difficult to define for benthic foraging pinnipeds, such as the AUFS, because they combine travel and searching, making it difficult to disentangle these components (Arnould & Hindell, 2001; Kirkwood & Arnould, 2011). As such, we have taken a conservative approach when calculating prey profitability.

### Statistical analyses

All statistical analyses were performed in the R statistical environment (version 3.6.1) (R Core Development Team, 2019). Data exploration was conducted following the protocols described in Zuur, Ieno & Elphick (2010). Based on initial exploratory analyses and the nested nature of the data, mixed models, with a random effect of ‘year’, were used to account for differences in sampling effort between years. To assess the influence of prey type on pursuit times, handling times, and gross energy intake, linear mixed effects models (LME) were constructed using the *lme4* package (Bates et al., 2015). Log transformations were applied to pursuit times, handling times, and gross energy intake, to approximate a normal distribution of residuals. Prior to modelling, pursuit and handling times were subset to include only successful prey captures. Gross energy intake, total energy expenditure and prey profitability were only calculated for successful prey captures as it was not possible to obtain accurate estimates of prey size. To assess the influence of prey type on energy expenditure and prey profitability, generalised mixed effects models (GLMM) constructed with a Gamma distribution using the *lme4* package. All results are reported as medians unless stated otherwise.

## Results

### Prey types and capture success

Video data were obtained from a total of 23 individuals, totalling 84.5 h (Table S2). A total of 2,027 prey detection events were observed in the video data, with 2,022 leading to prey pursuits (99.7%) and 1,263 observed prey captures (62.3%). The main identifiable prey types observed were Scorpaeniformes (gurnards, gurnard perches, and flatheads; 28.8%), Carangiformes (jack mackerel *Trachurus spp.* and trevally *Pseudocaranx* spp.; 15.1%), and Tetraodontiformes (leatherjackets; 12.3%) (Table 1). All other identifiable prey types contributed to <2.0% of the observed prey, while a total of 39.3% of observed prey (all benthic) could not be identified. Unidentified (*i.e.*, ‘Benthic — Unknown’) prey captures and prey capture attempts predominantly occurred at night.

For the detected prey, the highest capture successes (100%) were for benthic cephalopods and solitary fish (leatherjackets) detected in the pelagic zone (Table 2). Solitary fish detected at the seafloor (*e.g.*, gurnards and leatherjackets) and elasmobranchs were successfully captured 85.8% and 62.5% of detections, respectively. Baitfish encountered in both pelagic and demersal zones had the lowest prey capture success (32.9% and 39.4%, respectively). Unknown benthic prey items were captured with a 44.8% success rate, comparable to that of demersal baitfish.

**Table 1:** Number of prey events recorded through animal-borne cameras attached to 23 female Australian fur seals in northern Bass Strait, south-eastern Australia, during the austral winter (May–August) between 2008–2017.

**Prey type**	**Encountered (n)**	**Captured (n)**	**Proportion of items (%)**
**Invertebrates**			
Cephalopoda - Giant cuttlefish (*Sepia apama*)	1	1	0.08
Cephalopoda - Octopoda (*Octopus* spp.)	30	30	2.38
Cephalopoda - Tethida (squids)	5	5	0.40
Other - Spiny rock lobster (*Jasus edwardsii*)	1	1	0.08
**Vertebrates**			
Elasmoranchii - Carcharciniformes (catsharks)	1	0	0.00
Elasmoranchii - Myliobatiformes (stingrays)	8	8	0.63
Elasmoranchii - Necklace carpetshark (*Parascyllium variolatum*)	1	0	0.00
Elasmoranchii - Squaliformes (dogfishes)	1	0	0.00
Beloniformes - Garfish (*Hyporhamphus* spp.)	2	2	0.16
Carangiformes - Jack Mackerel (*Trachurus* spp.)	280	85	6.73
Carangiformes - Trevally (*Pseudocaranx* spp.)	5	3	0.24
Carangiformes - Other Carangidae	21	11	0.87
Gadiformes - Codling (Moridae)	6	4	0.32
Ophidiiformes - Pink Ling (*Genypterus blacodes*)	3	3	0.24
Perciformes - Knifejaw (*Oplegnathus* spp.)	1	1	0.08
Perciformes - Redbait (*Emmelichthys nitidus*)	4	3	0.24
Scombriformes - Barracouta (*Thyrsites atun*)	3	3	0.24
Scorpaeniformes - Neosebastidae (gurnard perches)	83	75	5.94
Scorpaeniformes - Platycephalidae (flatheads)	6	5	0.40
Scorpaeniformes - Triglidae (gurnards)	334	301	23.83
Scorpaeniformes –Other	160	119	9.42
Tetraodontiformes - Monacanthidae (leatherjackets)	246	225	17.81
Tetraodontiformes - Slender-spined porcupine fish (*Diodon nichthemerus*)	3	3	0.24
Zeiformes - Silver dory (*Cyttus australis*)	24	18	1.43
**Unidentified**			
Unidentified benthic prey	793	357	28.27
**Total**	2022	1263	

**Table 2:** Success rate for prey capture attempts in the dominant prey groups of the Australian fur seal (*Arctocephalus pusillus doriferus*) diet as determined by animal-borne video.

**Prey type**	**Pursuit initiations (n)**	**Captures** **(n)**	**Success rate (%)**
Benthic - Cephalopods	34	34	100.0
Benthic - Elasmobranchs	16	10	62.5
Benthic - Solitary fish	786	674	85.8
Benthic - Unknown	783	351	44.8
Demersal - Baitfish	66	26	39.4
Pelagic - Baitfish	234	77	32.9
Pelagic - Solitary fish	85	85	100.0

### Prey pursuit and handling times

**Figure 2:**
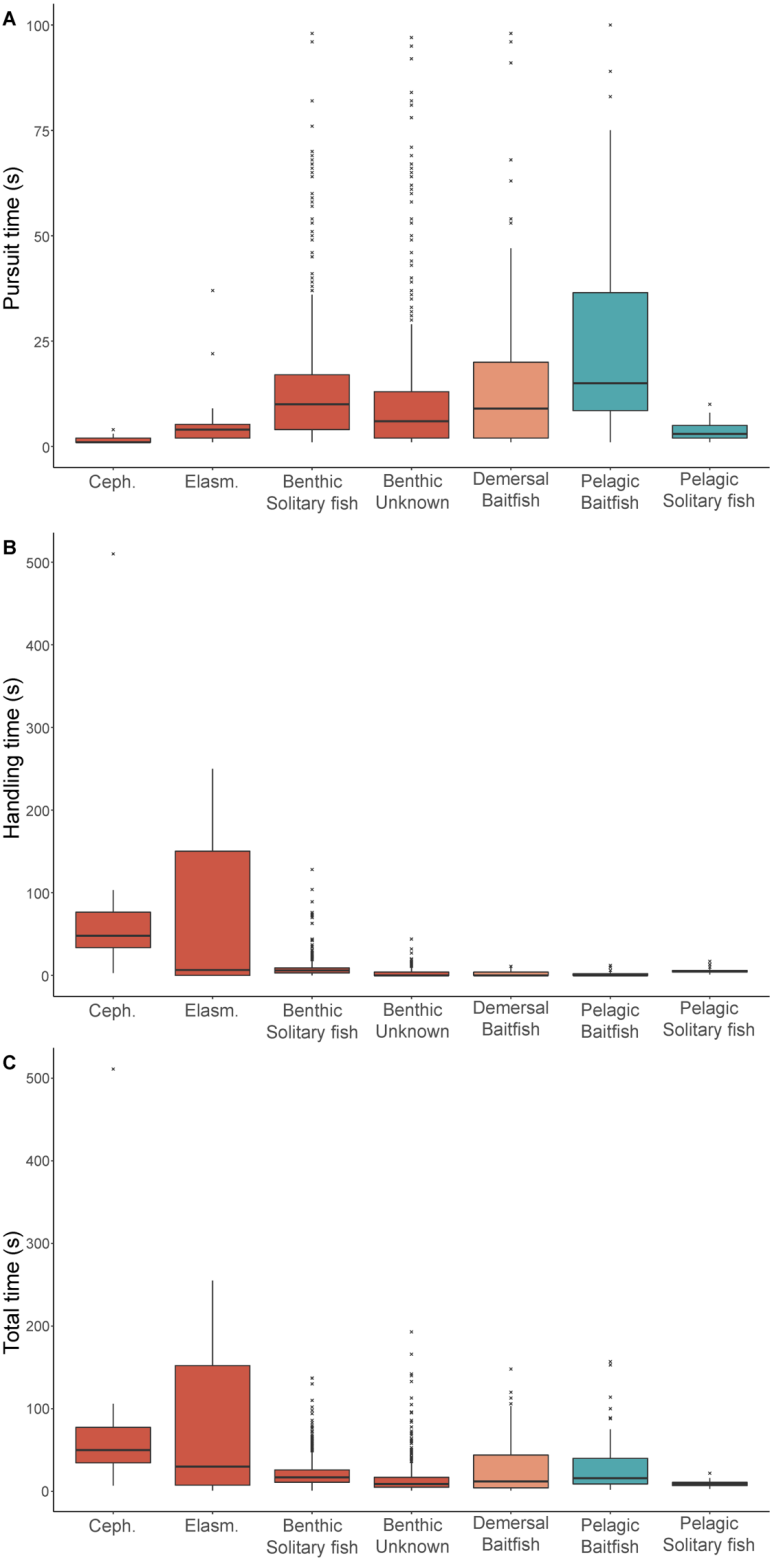
Prey pursuit and handling times for female Australian fur seals. Boxplots comparing the (A) pursuit time, (B) handling time, and (C) total time spent by female Australian fur seals on individual prey captures for different prey types in Bass Strait, south-eastern Australia. Ceph. = cephalopod, Elasm. = elasmobranch. Benthic prey are shown in red, demersal prey in orange, and pelagic prey in green. The median value is indicated by the thick horizontal line. Upper and lower bounds of the boxes represent the 75th and 25th percentiles, respectively, and the interquartile range (IQR) is the difference between the 25th and 75th percentiles. Whiskers represent 1.5*IQR. Any values extending beyond the whiskers are considered outliers (grey dots). Sample sizes are indicated in Table 1 (pursuit time) and Table 2 (handling and total time).

For prey that could be identified, both pursuit and handling times were significantly influenced by prey type (Table 3). While median pursuit times for cephalopods (1 s) and elasmobranchs (4 s) were short, median handling times for these prey types were the highest (55 s and 139 s, respectively) (Fig. 2). With the exception of pelagic solitary fish, all other prey types had longer pursuit times than handling times. Baitfish in the pelagic foraging zone required longer pursuit times (8 s) than in the demersal zone (4 s), while handling times were very short (<1 s) for both. Similarly, pursuit times were longer for solitary fish detected in the pelagic zone than near the seafloor (7 s and 4 s, respectively). However, handling times were higher for solitary fish captured in the benthic zone (6 s) than the pelagic zone (5 s). This may reflect the differences in the species consumed, with solitary fish consumed in the pelagic zone comprised mostly of leatherjackets while in the benthic zone gurnards were the dominant item. Unknown benthic prey had a median pursuit and handling times of 4 s and 1 s, respectively.

**Table 3:** Summary for the Linear Mixed Effects (LME) models used to identify the influence of prey type on the pursuit time, handling time, gross energy intake, total energy expenditure and prey profitability for female Australian fur seals instrumented with video data loggers during the austral winter (May–August) between 2008–2017. The reference prey group is ‘Benthic – Cephalopods’.

**Model**	**Covariates**	**Est.**	**SE**	***t* value**	**Pr (>∣*t*∣)**
log(Pursuit time) ∼ Prey type	(Intercept)	3.73	0.19	20.15	<0.001
	Benthic - Elasmobranchs	0.77	0.32	2.40	0.017
	Benthic - Solitary fish	−1.82	0.18	−9.95	<0.001
	Benthic - Unknown	−2.11	0.18	−11.46	<0.001
	Demersal - Baitfish	−1.85	0.34	−5.38	<0.001
	Pelagic - Baitfish	−2.84	0.27	−10.51	<0.001
	Pelagic - Solitary fish	−1.94	0.24	−7.90	<0.001
log(Handling time) ∼ Prey type	(Intercept)	0.48	0.21	2.26	0.024
	Benthic - Elasmobranchs	0.43	0.32	1.37	0.172
	Benthic - Solitary fish	1.04	0.20	5.33	<0.001
	Benthic - Unknown	0.68	0.20	3.47	0.001
	Demersal - Baitfish	0.69	0.25	2.80	0.005
	Pelagic - Baitfish	1.92	0.23	8.50	<0.001
	Pelagic - Solitary fish	0.63	0.25	2.58	0.010
log(Energy gain) ∼ Prey type	(Intercept)	7.31	0.26	28.53	<0.001
	Benthic - Elasmobranchs	−2.05	0.37	−5.59	<0.001
	Benthic - Solitary fish	−1.29	0.27	−4.81	<0.001
	Demersal - Baitfish	−0.54	0.33	−1.66	0.099
	Pelagic - Baitfish	−0.74	0.27	−2.69	0.009
	Pelagic –Solitary fish	−1.43	0.32	−4.53	<0.001
log(Energy expended) ∼ Prey type	(Intercept)	3.17	0.29	10.98	<0.001
	Benthic - Elasmobranchs	−0.15	0.41	−0.37	0.712
	Benthic - Solitary fish	−0.94	0.31	−3.11	0.003
	Demersal - Baitfish	−0.14	0.37	−0.38	0.702
	Pelagic - Baitfish	0.46	0.31	1.48	0.142
	Pelagic –Solitary fish	−1.83	0.36	−5.13	<0.001
log(Profitability) ∼ Prey type	(Intercept)	2.98	0.30	9.88	<0.001
	Benthic - Elasmobranchs	−1.85	0.42	−4.39	<0.001
	Benthic - Solitary fish	−0.18	0.31	−0.59	0.555
	Demersal - Baitfish	0.14	0.37	0.37	0.711
	Pelagic - Baitfish	0.20	0.31	0.63	0.533
	Pelagic –Solitary fish	0.72	0.36	1.98	0.051

### Energy gain and prey profitability

**Figure 3:**
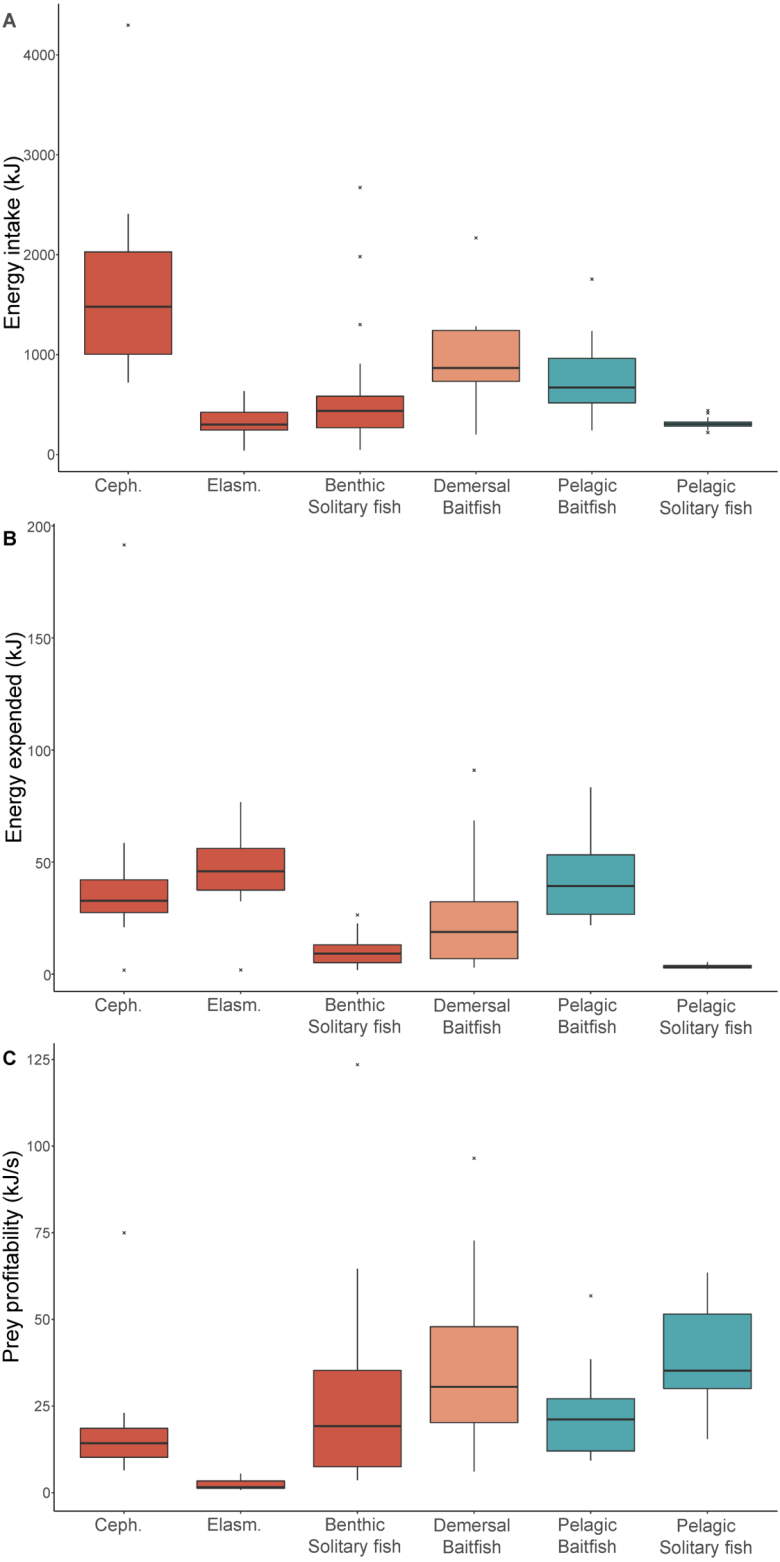
Diet energetics of female Australian fur seals. Boxplots comparing (A) gross energy intake, (B) total energy, and (C) prey profitability of individual prey captures for different prey type groups captured by female Australian fur seals in Bass Strait, south-eastern Australia. Ceph. = cephalopod, Elasm. = elasmobranch. Benthic prey are shown in red, demersal prey in orange, and pelagic prey in green. The median value is indicated by the thick horizontal line. Upper and lower bounds of the boxes represent the 75th and 25th percentiles, respectively, and the interquartile range (IQR) is the difference between the 25th and 75th percentiles. Whiskers represent 1.5*IQR. Any values extending beyond the whiskers are considered outliers (grey dots). Sample sizes are indicated in Table 2.

The median length and mass estimates of the main prey types consumed ranged 14–33 cm and 87–798 g, respectively (Table 4). Prey type had a significant influence on net energy intake, with energy intake from cephalopods (the reference group) significantly higher than elasmobranchs (Est = −2.1, *t* =  − 5.6, *p* < 0.001), benthic solitary fish (Est = −1.3, *t* =  − 4.8, *p* < 0.001), pelagic solitary fish (Est = −1.4, *t* =  − 4.5, *p* < 0.001), and pelagic baitfish (Est = −0.7, *t* = 2.7, *p* = 0.009) but not significantly different from demersal baitfish (Est = −0.5, *t* = 1.7, *p* = 0.099) (Table 3). While the nutritional value of cephalopods (3.4 kJ g^−1^) was lower than that of all other prey types (Table S1), they provided the highest net energy intake (1,451 kJ) due to their greater biomass consumption (798 g). In contrast, despite their higher energy density, benthic solitary fish, pelagic baitfish, and demersal baitfish resulted in moderate net energy intake (431 kJ, 632 kJ, and 840 kJ, respectively). Elasmobranchs and pelagic solitary fish were of lower nutritional value (3.8 kJ g^−1^ and 3.5 kJ g^−1^, respectively), and, because these prey types were of low mass, they represented low net energy intake per item (237 kJ and 300 kJ, respectively) (Fig. 3).

**Table 4:** Estimates of the fork (fish), total (elasmobranch) and mantle (cephalopod) length and consumed mass of a representative sample of prey items consumed by female Australian fur seals in northern Bass Strait, south-eastern Australia. Median [range] values given for main prey types. Estimated consumed mass was determined from the calculated size, published gross energy density and proportion of the prey items consumed (baitfish and solitary fish 100%; elasmobranchs 78%; cephalopod 74%) from the video data.

**Prey type**	***n***	**Length (cm)**	**Estimated consumed mass (g)**
Benthic – cephalopods	8	14 [10–21]	798 [389–2318]
Benthic - elasmobranchs	8	33 [16–42]	162 [22–342]
Demersal - baitfish	10	23 [13–34]	131 [30–327]
Benthic - solitary fish	39	19 [9–35]	116 [10–566]
Pelagic - baitfish	20	21 [14–31]	101 [37–265]
Pelagic - solitary fish	19	16 [14–19]	87 [63–126]

Energy expenditure was also significantly influenced by prey type (Table 3). The energy expended was significantly lower for pelagic and benthic solitary fish (3.2 kJ and 9.2 kJ, respectively) compared to the reference group, benthic cephalopods (Est = −1.8, *t* =  − 5.1, *p* < 0.001 and Est = −0.9, *t* =  − 3.1, *p* = 0.003, respectively) (Fig. 3). All other prey types had energy expenditure that was not significantly different to cephalopods (Table 3). The energy expended to capture baitfish was considerably higher in the pelagic zone (39.3 kJ) than in the demersal zone (18.9 kJ), due to the longer pursuit times midwater. Benthic cephalopods and elasmobranchs were almost exclusively brought to the surface during the handling period to facilitate breakdown and consumption, which required a considerable amount of energy (32.8 kJ and 45.9 kJ, respectively).

The most profitable prey species for AUFS to capture and consume were solitary fish captured in the pelagic zone (35.2 kJ s^−1^), while solitary prey captured in the benthic zone was considerably lower profitability (19.2 kJ s^−1^) (Fig. 3). Consumption of baitfish was more profitable when prey were captured in the demersal zone than the pelagic zone (30.6 kJ s^−1^ and 21.2 kJ s^−1^, respectively) (Fig. 3). Cephalopods showed a non-significant trend toward lower profitability (14.3 kJ s^−1^) than solitary fish and baitfish (Table 3). Elasmobranchs were of significantly lower profitability (1.6 kJ s^−1^) than the benthic cephalopod reference group (Est = −1.7, *t* =  − 4.2, *p* < 0.001).

## Discussion

The present study used animal-borne video cameras to investigate the energetics of prey capture for female AUFS. Prey type related differences in the pursuit and handling times and, subsequently, the gross energy intake and expenditure during prey captures were identified. However, the profitability of prey did not vary significantly between most prey types due to the interplay of pursuit and handling dynamics.

### Prey types, pursuit and handling times

While individual foraging specialisations have been observed in AUFS (Kernaleguen et al., 2016), as a species they are considered opportunistic, generalist feeders (Gales & Pemberton, 1994). Consistent with previous diet studies (Deagle, Kirkwood & Jarman, 2009; Gales et al., 1993; Hume et al., 2004), video data in the present study indicated that AUFS consumed a wide range of known prey species. Individuals foraged mainly on Scorpaeniformes (gurnards and gurnard perches), which were detected in the benthic zone, and Carangiformes (jack mackerels) and Tetraodontiformes (leatherjackets), which were detected both in the pelagic and benthic zones. Cephalopods were also consumed but accounted for only *ca* 2% of the recorded items consumed, while all other prey types captured contributed minimally in overall proportion.

Whereas some previous diet studies have reported the occurrence of redbait (*Emmelichthys nitidus*) in high proportions (16–24%) of AUFS scat samples (Gales & Pemberton, 1994; Hume et al., 2004; Kirkwood, Hume & Hindell, 2008), redbait contributed to only 0.2% of the prey captures in the present study. However, there is strong inter-annual variation in AUFS diet (Kirkwood, Hume & Hindell, 2008), which may explain the discrepancies observed. The differences observed in the present study compared with previous diet analyses may also, in part, be due to the programming schedules of the cameras, switching on below 40 m depth, limiting the coverage of foraging trips and not providing data representative of the entire diet of the species (Kernaleguen et al., 2016).

Pursuit and handling times by female AUFS differed significantly between prey types. Pursuit times were high for baitfish in both pelagic and demersal foraging zones but were considerably higher within the pelagic zone. Baitfish, such as jack mackerels, are often encountered in bait balls in the mid-water (Keenleyside, 1955). The schooling behaviour is an antipredator strategy, reducing the predation risk of each individual through a dilution and confusion effect on the predator (Hager & Helfman, 1991; Romano et al., 2020). This can lead to increasing pursuit times and reducing capture success for marine predators (Crook & Davoren, 2014; Dänhardt & Becker, 2011). The greater manoeuvrability of baitfish (due to their substantially smaller size) than AUFS (Blake & Chan, 2006), also impedes easy prey capture and, consequently, increases pursuit times (Video S1). The reduced pursuit times for baitfish in the demersal zone may be due to baitfish being trapped by the seafloor, making capture easier than when baitfish school mid-water. All other prey types had considerably lower pursuit times, reflecting the easier capture of solitary prey that has been observed for other pinnipeds (Bowen et al., 2002).

Handling times for most fish species were very short (<6 s), with prey consumed whole, enabling individuals to continue hunting and capture multiple prey within the same dive. However, due to their large size, handling times were substantially longer for elasmobranchs and cephalopods, with these items typically being brought to the surface for processing and consumption (Video S2). Similarly, larger solitary fish were occasionally handled at the surface. This is consistent with other predator studies reporting longer handling times for larger prey items (Brisson-Curadeau & Elliott, 2019). This relationship between handling time and prey size is a major driver of the reduced variability in net energy gain between different prey types (Stephens et al., 1986).

The pursuit and handling times, and the influence of prey type of pursuit and handling times, may be somewhat impacted by potential drag effects from the video cameras. Several devices were used throughout the study which had varying sizes and shapes, though similar frontal area. These drag effects may have resulted in pursuit and handling times that were greater than they would have been in the absence of devices. Animal-borne video cameras have been associated with changes in diving behaviour of lactating female Antarctic fur seals even when weighing <1.5% of the seal’s body mass (Heaslip & Hooker, 2008). Since we are interested in the relative, rather than exact, profitability of prey, any drag effects were unlikely to impact these relative values.

### Energy gain and prey profitability

While the results of the present study revealed substantial differences in gross energy content between prey species consumed by adult female AUFS, there was little variation in the energetic profitability of most prey types. Despite their low gross energy density, cephalopods represented the highest energy intake per prey item due to their larger size and low pursuit times. However, the short pursuit times were confounded by the need to process cephalopods at the surface. This increased handling durations, which, when combined with incomplete consumption, resulted in cephalopods having relatively low energetic profitability to adult female AUFS. Elasmobranchs also had relatively short pursuit times. However, similar to cephalopods, elasmobranchs were the least profitable AUFS prey due to their long handling times and incomplete consumption.

Baitfish had markedly higher energy density than all other AUFS prey species recorded in the present study. However, the small size of baitfish resulted in lower energy intake in comparison to cephalopods. Furthermore, while the handling duration of baitfish was short, pursuit times were typically long, which resulted in higher energy expenditure and average energetic profitability. Solitary fish did not constitute high energy intake for individuals due to their small size but their short pursuit and handling durations resulted in low energy expenditures. In particular, pelagic solitary fish represented some of the most profitable prey because of short pursuit and handling durations. Indeed, this prey type was comprised almost entirely of leatherjackets (Tetraodontiformes) that were mostly captured unsuspectingly from below by individuals (Video S3). While not forming schools, leatherjackets occurred in loose aggregations with multiple individuals being captured within single dives which could further enhance the net energy gain from this prey type. However, the availability and distribution of such aggregations might vary their encounter rate between seasons or years (Speakman et al., 2020). Therefore, when search times are accounted for, benthic prey may provide greater overall profitability.

Not all prey types recorded were consumed by all AUFS adult females in the present study, such that it was not possible to assess the influence of body size on pursuit and handling time and, thus, on profitability of the different prey. However, previous studies have revealed influences of body size on trophic niche and specialisation (Arnould et al., 2011; Kernaleguen et al., 2016). Correspondingly, the profitability of different prey types observed in the present study could reflect specialisations in the pursuit, capture and handling of specific prey types by the AUFS adult females instrumented with video data loggers. Individuals may prefer and specialize on prey they are efficient at handling and consuming, while ignoring other prey. While AUFS have been found to be largely generalist foragers, diet specialization has been observed in some individuals (Kernaleguen et al., 2016). Hence, the estimated profitability of prey items observed in the present study may not be representative for all adult female AUFS (Tinker et al., 2012).

Indeed, as it was not possible to assess the size and, thus, energy content of prey in unsuccessful capture attempts, the present study only analysed profitability of successful captures. The exclusion of unsuccessful prey capture attempts may have biased the profitability of prey calculated in this study. The lowest capture success rates were observed for schooling baitfish (<40%) and, correspondingly, if the energetic costs of abandoned pursuits were accounted for, the overall apparent profitability of these prey types would be substantially lower (and likely the lowest of all prey types). Australian fur seals are considered to be almost exclusively benthic foragers (Arnould & Hindell, 2001; Hoskins & Arnould, 2014), suggesting pelagic schooling baitfish do not constitute a large proportion of their diet and may be detected at a lower rate than benthic prey items. Furthermore, it has been suggested the large body size of AUFS prevents them exploiting small pelagic schooling fish prey unless they are in very high abundance (Arnould & Costa, 2006). A recent long-term multi-year study of dive behaviour and foraging success in AUFS revealed fluctuations in the proportion of pelagic dives which were related to environmental variability (Speakman et al., 2020). These observations could indicate that, under certain environmental conditions, the abundance of pelagic schooling fish makes them energetically worthwhile for AUFS to hunt, implying AUFS could use a range of benthic and pelagic foraging strategies, depending on prey availability.

While the present study revealed similarities in the net rate of energy gain of prey in terms of their pursuit/handling costs, search times are normally included when determining the overall profitability of prey and its influence on optimal foraging decisions by individuals (Engen & Stenseth, 1984; Stephens et al., 1986). However, defining search times in benthic foraging pinnipeds, such as AUFS, is difficult as it requires the disentanglement of search time from commute and predator avoidance (Arnould & Hindell, 2001; Jewell et al., 2019). As a result, the profitability estimates in the present study may under- or over-estimate the profitability of prey that require shorter/longer search times.

The demersal and benthic prey items targeted by AUFS are typically more ubiquitous across the seabed habitats within Bass Strait than the patchily distribution pelagic schooling fish and, consequently, may be more profitable due to the higher chance of encountering benthic and demersal prey (Arnould & Costa, 2006). Previous studies in otariid seals have attempted to infer profitability of benthic *versus* pelagic foraging strategies from at-sea movements, diving behaviour and accelerometry (Blakeway et al., 2021). However, their interpretations were limited due to a lack of information on the distribution, abundance, and community structure of the prey field. As technological advances continue to improve video data logger miniaturisation, memory storage and battery life capacity, future studies should aim to collect prey field information for complete foraging trips and under a variety of environmental conditions to better assess differences in prey profitability that may influence foraging decisions.

## Conclusion

In summary, the present study identified differences in the pursuit and handling times associated with the main prey types consumed by adult female AUFS. These differences impacted the total energy expenditure associated with prey acquisition and consumption. However, the different prey types had similar overall profitability. The results reinforce the importance of measuring the distribution, abundance and community structure of prey to better understand the factors that influence foraging decisions in predators and highlight the benefit of animal-borne video data loggers for such endeavours. Future studies should investigate levels of individual variation in pursuit and handling times and elucidate how individual hunting strategies impact profitability of different prey types.

## Supplemental Information

10.7717/peerj.12608/supp-1 Supplemental Information 1 Energetic values of representative prey types of each group used to estimate the nutritional content of prey observed in the study

10.7717/peerj.12608/supp-2 Supplemental Information 2 Summary of morphometric measurements for 23 female Australian fur seals instrumented with a video data logger in central-northern Bass Strait, south-eastern Australia, during the austral winter (May–August) between 2008–2017

10.7717/peerj.12608/supp-3 Supplemental Information 3 Adult female Australian fur seal chasing jack mackerel Video footage is from an animal-borne video camera deployed by J.P.Y. Arnould.

10.7717/peerj.12608/supp-4 Supplemental Information 4 Adult female Australian fur seal capturing octopus and bringing to the surface Video footage is from an animal-borne video camera deployed by J.P.Y. Arnould. Following capture, 44 s of video was removed as the female ascended through the water column to the surface. Note: date/time stamp incorrect.

10.7717/peerj.12608/supp-5 Supplemental Information 5 Adult female Australian fur seal capturing leatherjackets Video footage is from an animal-borne video camera deployed by J.P.Y. Arnould.

## Additional Information and Declarations

### Field Study Permissions

The following information was supplied relating to field study approvals (i.e., approving body and any reference numbers): Department of Land, Water Environment and Planning (Victoria, Australia) Wildlife Research Permits (10005484, 10007153, 1000826).

### Data Availability

The following information was supplied regarding data availability: The data are available at Zenodo: Nelle Meyers, Cassie S. Speakman, Nicole AS-Y Dorville, Mark A. Hindell, Jayson Semmens, Jacquomo Monk, Alistair M.M. Baylis, Daniel Ierodiaconou, Andrew J. Hoskins, Greg J. Marshall, Kyler Abernathy, & John P.Y. Arnould. (2021). Data set for paper on Australian fur seal prey capture and foraging efficiency [Data set]. Zenodo. https://doi.org/10.5281/zenodo.5598295.
