# Supplementary material for: The cost of a meal: factors influencing prey profitability in Australian fur seals
Source: PeerJ. 2021 Dec 8;9:e12608. doi: 10.7717/peerj.12608 (PMC8667761; doi:10.7717/peerj.12608)
Supplement: Supplemental Information 1 [file peerj-09-12608-s001.docx]

**Electronic Supplementary Material:**

The cost of a meal: factors influencing prey profitability in Australian fur seals

Nelle Meyers, Cassie N. Speakman, Nicole A. S.-Y. Dorville, Mark A. Hindell, Jayson Semmens, Jacquomo Monk, Alistair M. M. Baylis, Daniel Ierodiaconou, Andrew J. Hoskins, Greg J. Marshall, and Kyler Abernathy and John P. Y. Arnould

Corresponding author: john.arnould@deakin.edu.au (JPYA)

**Table S1**. Energetic values of representative prey types of each group used to estimate the nutritional content of prey observed in the study.

| **Prey type** | **Energetic value (kJ**·**g^-1^)** | **References** |
| --- | --- | --- |
| Benthic - cephalopods (octopus spp.) | 3.4 | (Alejo-Plata & Mendez 2014; Pehrsson et al. 2015) |
| Benthic - elasmobranchs (stingray spp.) | 3.8 | (Lteif et al. 2016; Sidwell 1981) |
| Demersal/Pelagic - baitfish (mackerel spp.) | 6.6 | (Sidwell 1981; Smallwood et al. 2017) |
| Benthic/Pelagic - solitary fish (leatherjacket spp.) | 3.5 | (Sidwell 1981; Smallwood et al. 2017) |
| Benthic - solitary fish (Scorpaeniformes) | 4.2 | (Pehrsson et al. 2015; Vallisneri et al. 2010) |

Alejo-Plata MD, and Mendez OV. 2014. Arm abnormality in *Octopus hubbsorum* (Mollusca: Cephalopoda: Octopodidae). *American Malacological Bulletin* 32:217-219. <https://doi.org/10.4003/006.032.0212>

Lteif M, Mouawad R, Jemaa S, Khalaf G, Lenfant P, and Verdoit-Jarraya M. 2016. The length-weight relationships of three sharks and five batoids in the Lebanese marine waters, eastern Mediterranean. *The Egyptian Journal of Aquatic Research* 42:475-477.

Pehrsson P, Patterson K, Haytowitz D, and Phillips K. 2015. Total carbohydrate determinations in USDA's national nutrient database for standard reference. *Faseb Journal* 29:1.

Sidwell VD. 1981. *Chemical and nutritional composition of finfishes, whales, crustaceans, mollusks, and their products*. Seattle, Washington: U.S. Dept. of Commerce, National Oceanic and Atmospheric Administration, National Marine Fisheries Service.

Smallwood CB, Tate A, and Ryan KL. 2017. *Weight-length summaries for Western Australian fish species derived from surveys of recreational fishers at boat ramps*: Fisheries Division, Department of Primary Industries and Regional Development.

Vallisneri M, Montanini S, and Stagioni M. 2010. Length-weight relationships for the family Triglidae in the Adriatic Sea, northeastern Mediterranean. *Journal of Applied Ichthyology* 26:460-462. <https://doi.org/10.1111/j.1439-0426.2009.01389.x>
