# Supplementary material for: The cost of a meal: factors influencing prey profitability in Australian fur seals
Source: PeerJ. 2021 Dec 8;9:e12608. doi: 10.7717/peerj.12608 (PMC8667761; doi:10.7717/peerj.12608)
Supplement: Supplemental Information 2 [file peerj-09-12608-s002.docx]

**Electronic Supplementary Material:**

The cost of a meal: factors influencing prey profitability in Australian fur seals

Nelle Meyers, Cassie N. Speakman, Nicole A. S.-Y. Dorville, Mark A. Hindell, Jayson Semmens, Jacquomo Monk, Alistair M. M. Baylis, Daniel Ierodiaconou, Andrew J. Hoskins, Greg J. Marshall, and Kyler Abernathy and John P. Y. Arnould

Corresponding author: john.arnould@deakin.edu.au (JPYA)

**Table S2**. Summary of morphometric measurements for 23 female Australian fur seals instrumented with a video data logger in central-northern Bass Strait, south-eastern Australia, during the austral winter (May-August) between 2008-2017.

| **Seal** | **Deployment date** | **Video** | **Mass** | **Standard length** | **Flipper length** | **Axillary girth** |
| --- | --- | --- | --- | --- | --- | --- |
|  |  | **(h)** | **(kg)** | **(cm)** | **(cm)** | **(cm)** |
| 1 | 17/05/2008 | 0.3 | 98.0 | 171.0 | 45.5 | 108.0 |
| 2 | 19/05/2008 | 2.5 | 90.0 | 160.0 | 43.5 | 106.5 |
| 3 | 23/05/2008 | 5.5 | 85.5 | 157.0 | 45.5 | 101.0 |
| 4 | 28/05/2008 | 2.0 | 91.0 | 160.5 | 41.0 | 112.5 |
| 5 | 5/06/2009 | 2.5 | 80.0 | 150.0 | 41.5 | 106.0 |
| 6 | 5/06/2009 | 1.5 | 63.5 | 146.5 | 40.5 | 98.5 |
| 7 | 7/06/2009 | 2.8 | 81.5 | 156.5 | 43.5 | 112.0 |
| 8 | 23/07/2010 | 2.4 | 63.5 | 145.0 | 42.0 | 92.0 |
| 9 | 25/07/2010 | 2.5 | 84.5 | 158.0 | 45.0 | 103.0 |
| 10 | 25/07/2010 | 1.4 | 75.5 | 141.5 | 41.5 | 110.0 |
| 11 | 14/05/2011 | 3.3 | 88.5 | 166.0 | 48.0 | 100.0 |
| 12 | 14/05/2011 | 2.3 | 50.5 | 132.0 | 40.0 | 82.0 |
| 13 | 15/05/2011 | 4.7 | 54.5 | 136.0 | 40.5 | 85.0 |
| 14 | 15/05/2011 | 4.5 | 90.5 | 158.5 | 46.0 | 103.0 |
| 15 | 25/05/2011 | 4.0 | 88.0 | 161.5 | 47.5 | 103.0 |
| 16 | 11/06/2011 | 2.8 | 87.5 | 142.0 | 39.0 | 95.5 |
| 17 | 14/06/2011 | 2.8 | 55.5 | 159.0 | 42.5 | 104.5 |
| 18 | 17/05/2012 | 3.5 | 78.0 | 134.0 | 39.0 | 85.5 |
| 19 | 3/07/2015 | 3.2 | 66.5 | 144.0 | 42.0 | 99.0 |
| 20 | 2/06/2017 | 10.0 | 76.5 | 161.5 | 45.0 | 93.0 |
| 21 | 9/06/2017 | 12.0 | 79.5 | 157.5 | 43.5 | 104.0 |
| 22 | 13/08/2018 | 3.0 | 89.0 | 166.0 | 45.5 | 104.0 |
| 23 | 13/08/2018 | 5.0 | 76.5 | 153.0 | 42.0 | 101.0 |
